# NEW APPROACH TO STUDYING COLLECTIVE EFFECTS OF GENETIC FACTORS ON ALZHEIMER’S DISEASE

**DOI:** 10.1093/geroni/igz038.362

**Published:** 2019-11-08

**Authors:** Anatoliy Yashin, Deqing Wu, Konstantin Arbeev, Igor Akushevich, Arseniy Yashkin, Matt Duan, Eric Stallard, Svetlana Ukraintseva

**Affiliations:** 1 Duke University, Durham, North Carolina, United States; 2 Duke University, Durham, United States

## Abstract

Despite the wide recognition of the multifactorial nature of Alzheimer’s disease (AD), mechanisms of the collective influence of genetic and environmental factors on AD remain poorly understood. We used the Cardiovascular Health Study (CHS) and Framingham Heart Study (FHS) data to investigate the effects of genetic and gene-environment interactions on AD prevalence in the association analysis using logistic regression. Based on results of this analysis, we developed several new measures of integrated effects of SNPs, including for specific gene or group of genes, by constructing SNP-specific Interaction Polygenic Risk Scores (SIPRSs), Gene-specific Interaction Polygenic Risk Scores (GIPRS), and Trait-specific Interaction Polygenic Risk Scores (TIPRS), and tested them in the above data. We found strong interaction effects on AD among the SNPs in NRG3 gene and smoking, and among the SNPs in ATM and creatinine. In summary, we developed a new approach to measuring the collective impact of SNPs on complex traits, and discovered significant effects of the newly constructed SIPRS, GIPRS, and TIPRS on AD prevalence. The results of this study open a new opportunity of investigating the joint impacts of genetic and environmental factors on AD and other phenotypes of aging and health.

